# Voltage-Gated Ion Channels and the Variability in Information Transfer

**DOI:** 10.3389/fncel.2022.906313

**Published:** 2022-07-22

**Authors:** Rahul Kumar Rathour, Hanoch Kaphzan

**Affiliations:** Sagol Department of Neurobiology, University of Haifa, Haifa, Israel

**Keywords:** active dendrites, information transfer, global sensitivity analysis, variability, voltage-gated ion channels

## Abstract

The prerequisites for neurons to function within a circuit and be able to contain and transfer information efficiently and reliably are that they need to be homeostatically stable and fire within a reasonable range, characteristics that are governed, among others, by voltage-gated ion channels (VGICs). Nonetheless, neurons entail large variability in the expression levels of VGICs and their corresponding intrinsic properties, but the role of this variability in information transfer is not fully known. In this study, we aimed to investigate how this variability of VGICs affects information transfer. For this, we used a previously derived population of neuronal model neurons, each with the variable expression of five types of VGICs, fast Na^+^, delayed rectifier K^+^, A-type K^+^, T-type Ca^++^, and HCN channels. These analyses showed that the model neurons displayed variability in mutual information transfer, measured as the capability of neurons to successfully encode incoming synaptic information in output firing frequencies. Likewise, variability in the expression of VGICs caused variability in EPSPs and IPSPs amplitudes, reflected in the variability of output firing frequencies. Finally, using the virtual knockout methodology, we show that among the ion channels tested, the A-type K^+^ channel is the major regulator of information processing and transfer.

## Introduction

One of the important features of neurons within a homogenous population is that they express variability in their various physiological parameters, both intrinsic and extrinsic (Marder, 2011; Marder et al., 2014; Rathour and Narayanan, 2019; Goaillard and Marder, 2021). While there are multiple studies concerning homeostasis that accounted for this variability (Goldman et al., 2001; Taylor et al., 2009; Marder and Taylor, 2011; Rathour and Narayanan, 2012, 2014), studies on information encoding/transfer in face of variability are few (Padmanabhan and Urban, 2010, 2014; Tripathy et al., 2013). The general theme that has emerged from studies on homeostasis and variability is that variability of physiological parameters between neurons is critical for maintaining the homeostasis on multiple levels, both the single neuron and circuit functioning, serving the aim of information encoding (Goldman et al., 2001; Prinz et al., 2003, 2004; Taylor et al., 2009; Padmanabhan and Urban, 2010; Marder, 2011; Marder and Taylor, 2011; Tripathy et al., 2013; Padmanabhan and Urban, 2014; Rathour and Narayanan, 2014; Anirudhan and Narayanan, 2015; Srikanth and Narayanan, 2015; Rathour et al., 2016; Mittal and Narayanan, 2018; Mishra and Narayanan, 2019; Basak and Narayanan, 2020; Jain and Narayanan, 2020; Goaillard and Marder, 2021; Roy and Narayanan, 2021).

The role of variable expression of voltage-gated ion channels in maintaining homeostasis in neuronal physiology is well established, but how this variable expression of voltage-gated ion channels and homeostasis affect information encoding/transfer is not fully understood. Variability in voltage-gated ion channels is crucial for information encoding by enabling variability in input and output processing, thereby reducing spike train correlations and redundancy in the population of neurons (Padmanabhan and Urban, 2010, 2014). Hence, understanding the effects of intrinsic variability on neuronal responses and neuronal coding is essential. Furthermore, it raises the question of whether maintaining homeostasis occurs at the expense of robust information transfer or homeostasis itself brings about robust information transfer, which has not been answered.

In this study, we aimed to investigate the relations between the variability of intrinsic properties between neurons and their ability to encode information while maintaining the intrinsic homeostasis of their functional maps—a graded progression of physiologically relevant measurement along the spatial axis of the neuron. Specifically, we asked whether maintaining homeostasis occurs at the expense of robust information transfer or homeostasis itself brings about robust information transfer.

For this, we utilized previously derived CA1 neuronal models, which showed homeostasis of six coexistent functional maps and expressed variability in five voltage-gated ion channels (Rathour and Narayanan, 2014). We found that neurons in the model population displayed variability in mutual information transfer. Likewise, we found that the input of EPSPs and IPSPs showed huge variability in their amplitudes, due to the variability in the expression of voltage-gated ion channels, which was also reflected in output processing, as firing frequencies of model neurons similarly displayed huge variability. Finally, using virtual knockout models we show that the *A*-type K^+^ channel is the major regulator of these effects.

## Results

**Figure 1:**
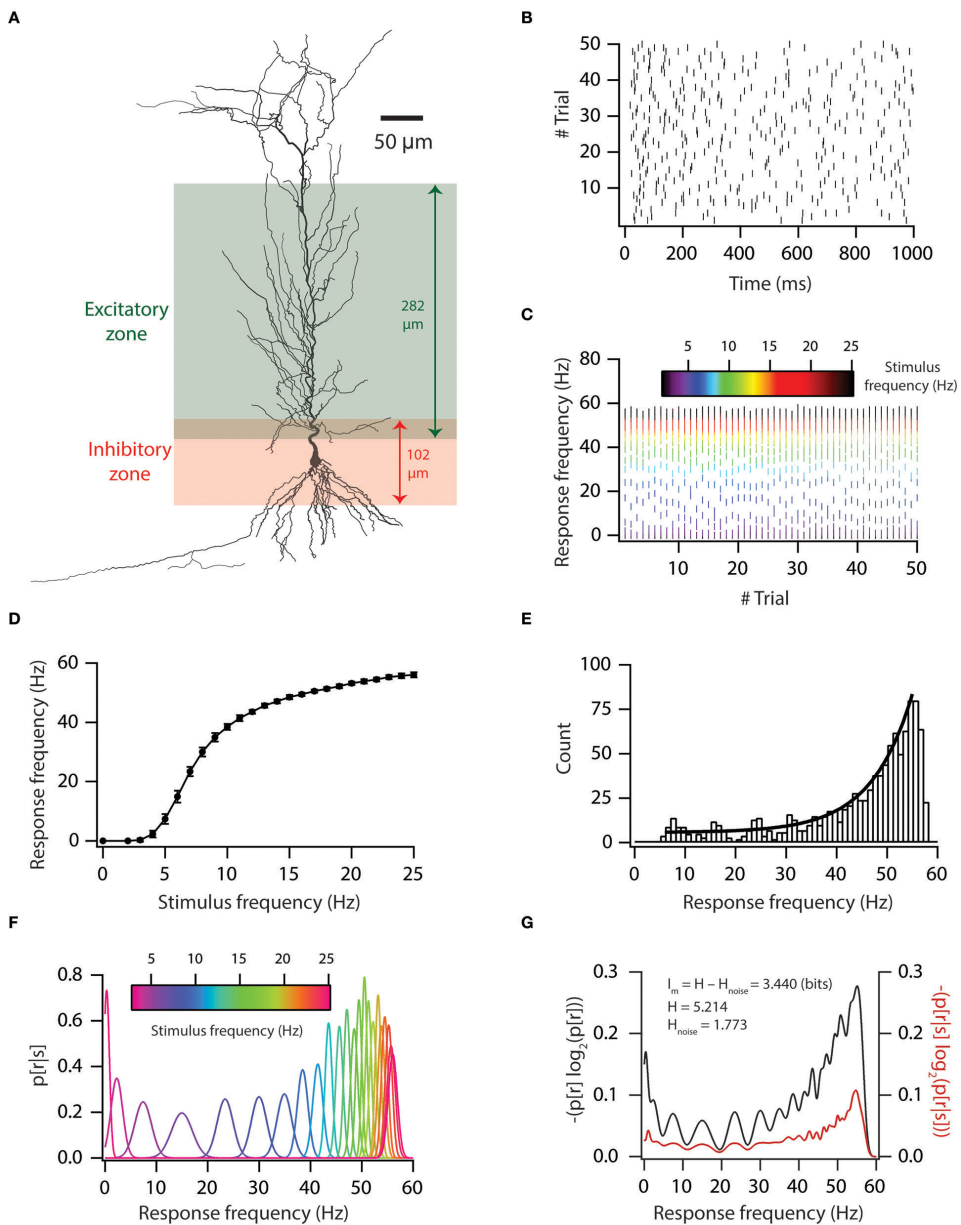
Input–output relationship and mutual information of base model. **(A)** A morphological reconstruction of CA1 pyramidal neuron used as a substrate for all simulations. **(B)** Trial-to-trial variability in spike times for 5-Hz Poisson-distributed inputs. **(C)** Trial-to-trial variability in firing frequency for different stimulus frequencies. **(D)** Input–output relationship in the base model. **(E)** Firing frequency distribution in the base model. Solid black line denotes the exponential fit. **(F)** Probability distribution of firing frequency, given stimulus frequency. **(G)** Graph showing the computation of mutual information from total response entropy and noise entropy for the base model.

We utilized a previously derived base model neuron and valid neuronal population (*n* = 228) (Rathour and Narayanan, 2014) to test how variability in voltage-gated ion channels (VGICs) expression in neurons affects robust information transfer. To this end, we first computed information encoding capabilities of a base model neuron (Figure 1), in which we employed a 3D reconstructed neuronal morphology (Figure 1A) expressing experimentally constrained six coexistent functional maps (Rathour and Narayanan, 2014), and included AMPA and GABA_A_ receptor-type excitatory and inhibitory synapses (Figure 1A). To reduce the computational cost, we uniformly distributed an excitatory synapse only on the apical side within a distance of 303 μm from the soma, while inhibitory synapses were placed in the perisomatic region within a distance of 50 μm from soma (Figure 1A). This led to a total of 327 excitatory synapses and 50 inhibitory synapses. We activated these synapses in a Poisson manner at different frequencies. This led to variability in inputs.

This variability in inputs led to variable output (Figures 1B,C). We also generated an input–output curve for the base model (Figure 1D). From the input–output curve, we noted that firing frequencies were exponentially distributed (Figure 1E). Under rate coding schema, mutual information was computed as the ability of the model neuron to successfully encode incoming information (different stimulus frequencies) in distinct neuronal outputs (firing frequencies). To do this, we computed response and noise entropy from neuronal inputs and outputs (Figures 1F,G). Then, we subtracted noise entropy from response entropy to obtain mutual information (Figure 1G). We found that a base model neuron had good information encoding capabilities (Figure 1G).

### Validation of Model Population Using *F*–*I* Relationship

The previously derived model population was validated with respect to the experimental data of six functional maps (Rathour and Narayanan, 2014). Here, we intended to test the information encoding capabilities of the model population neurons under the rate coding schema. At first, we aimed to show that the model population is truly representative of real-world neurons. To show this, we tested the *F*–*I* relationship of the model population to that of the experimental counterpart. For that, we generated the *F*–*I* relationship of the model population by injecting current from 0 to 250 pA in steps of 50 pA for 1 s and counted the number of spikes elicited, which provided us with various firing rate profiles of the neurons. Comparisons of these firing rates against the experimental data demonstrated that firing rates were largely within the experimentally observed ranges (Supplementary Figure S1) [experimental data taken from Narayanan and Johnston (2007); Narayanan et al. (2010); Rathour et al. (2016)], with no statistically significant differences. It is noteworthy that these models were never optimized for firing rate profiles, further proving the validity of our model population.

### Variability in Voltage-Gated Ion Channel Expression Causes Variable Input Processing

In order to study the role of variable VGIC expression in input processing, we first hand-tuned synaptic permeability values in the base model so that each excitatory synapse had unitary EPSP (uEPSP) amplitude at the soma of about 0.2 mV, irrespective of the synapse location (Supplementary Figure S2A). This ensured that a dendritic democracy (Magee and Cook, 2000) was maintained. Similarly, inhibitory synapses were fixed for uIPSP amplitude at the soma of about −1 mV (Supplementary Figure S2B).

**Figure 2:**
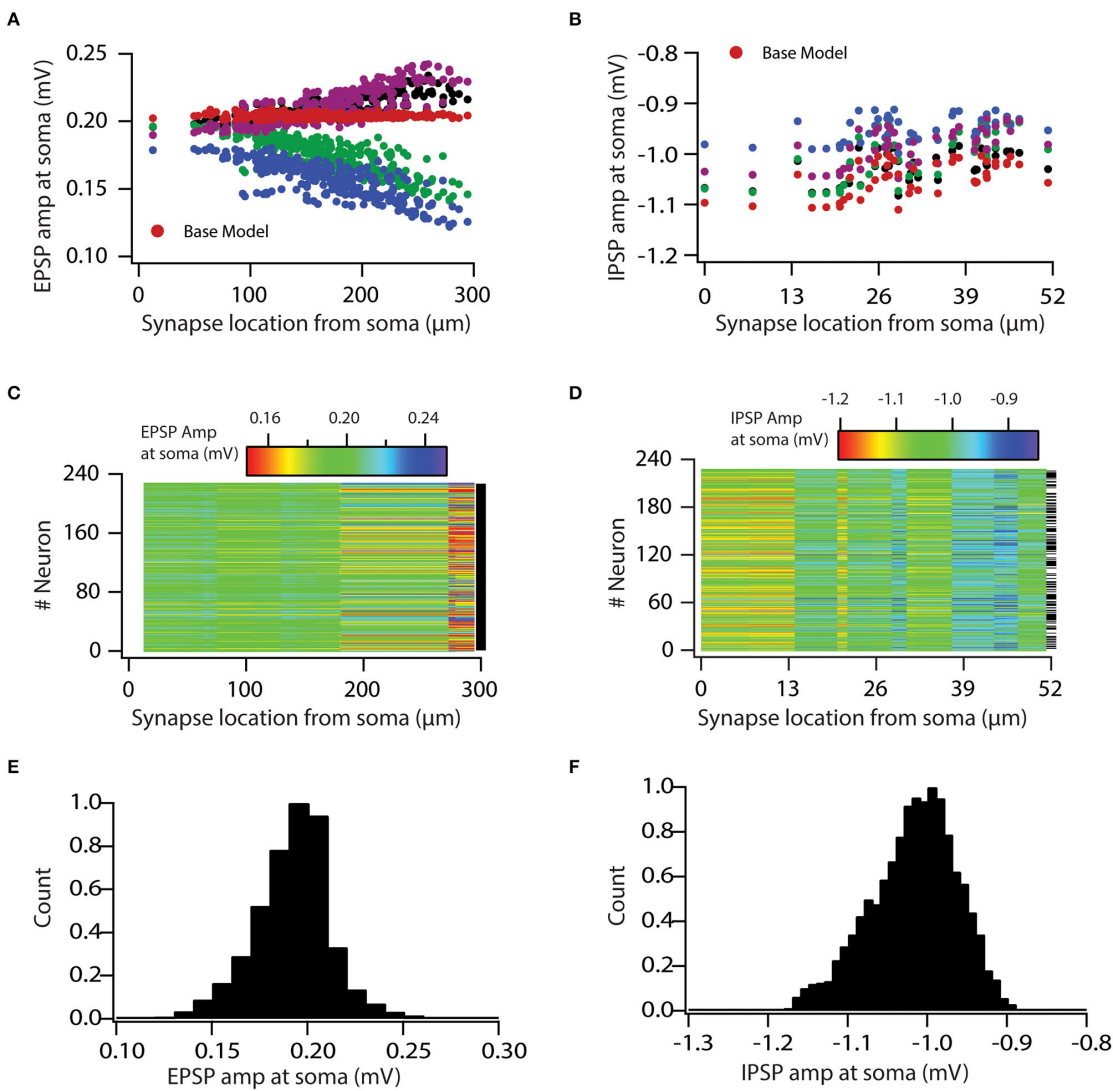
Variability in expression of functional maps leads to variable input processing in neuronal population. **(A,B)** uEPSP **(A)** and uIPSP **(B)** amplitudes at the soma as a function of synapse location for four valid model neurons and the base model neuron. **(C,D)** Color-coded uEPSP **(C)** and uIPSP **(D)** amplitudes at the soma as a function of synapse location for all the valid neurons. **(E,F)** Distribution of all uEPSPs **(E)** and uIPSPs **(F)** amplitudes in valid neuronal population. Note that conductance values of the given type of synapse are same for all the model neurons.

Next, in order to examine how variability in VGICs expression affects the variable input processing, we placed the same synapses with the same permeability values on a valid neuronal population, which generated somatic uEPSP and uIPSP amplitude maps (Figures 2A,B) for the entire valid neuronal population. As expected, we found that variability in VGICs expression and passive membrane properties induced variability in somatic uEPSP and uIPSP amplitude maps (Figure 2). Following, we examined whether somatic uEPSP and uIPSP amplitude maps of a valid neuronal population are significantly different from the base model neuron. KS tests between each pair of base model neuron and neuron of the valid neuronal population (total 228 pairs) showed that somatic uEPSP amplitude maps of the valid neuronal population were significantly different from those of the base model neuron for all pairs (Figure 2C; black dash against color-coded uEPSP amplitude maps denotes significance). Similarly, for somatic uIPSP amplitude maps of the valid neuronal population, we found that out of 228 pairs of neurons 122 pairs were significantly different from uIPSP amplitude map of the base model neuron (Figure 2D; black dash against color-coded uIPSP amplitude maps denotes significance). The *p*-value for performing the KS test was set at 0.001.

Following, we aimed to determine the boundaries for this variability and whether it is in a physiological range. For that, we generated histograms of all somatic uEPSP and uIPSP amplitudes in response to a single-synapse activation. This simulation yielded a rather large variability in the somatic uEPSP and uIPSP amplitudes (Figures 2E,F). Specifically, somatic uEPSP amplitude exhibited about ±25% variability in their amplitudes from the mean value of 0.19 mV, whereas somatic uIPSP amplitude exhibited about +12 and −10% variability in their amplitudes from the mean value of −1.01 mV. Nonetheless, somatic uEPSP and uIPSP amplitudes were still within physiological ranges. Taken together, these analyses clearly demonstrated the role of VGICs in input processing and showed that variability in VGICs expression leads to variable input processing.

### Variability in Neuronal Outputs Leads to Decorrelation in Firing Frequencies

**Figure 3:**
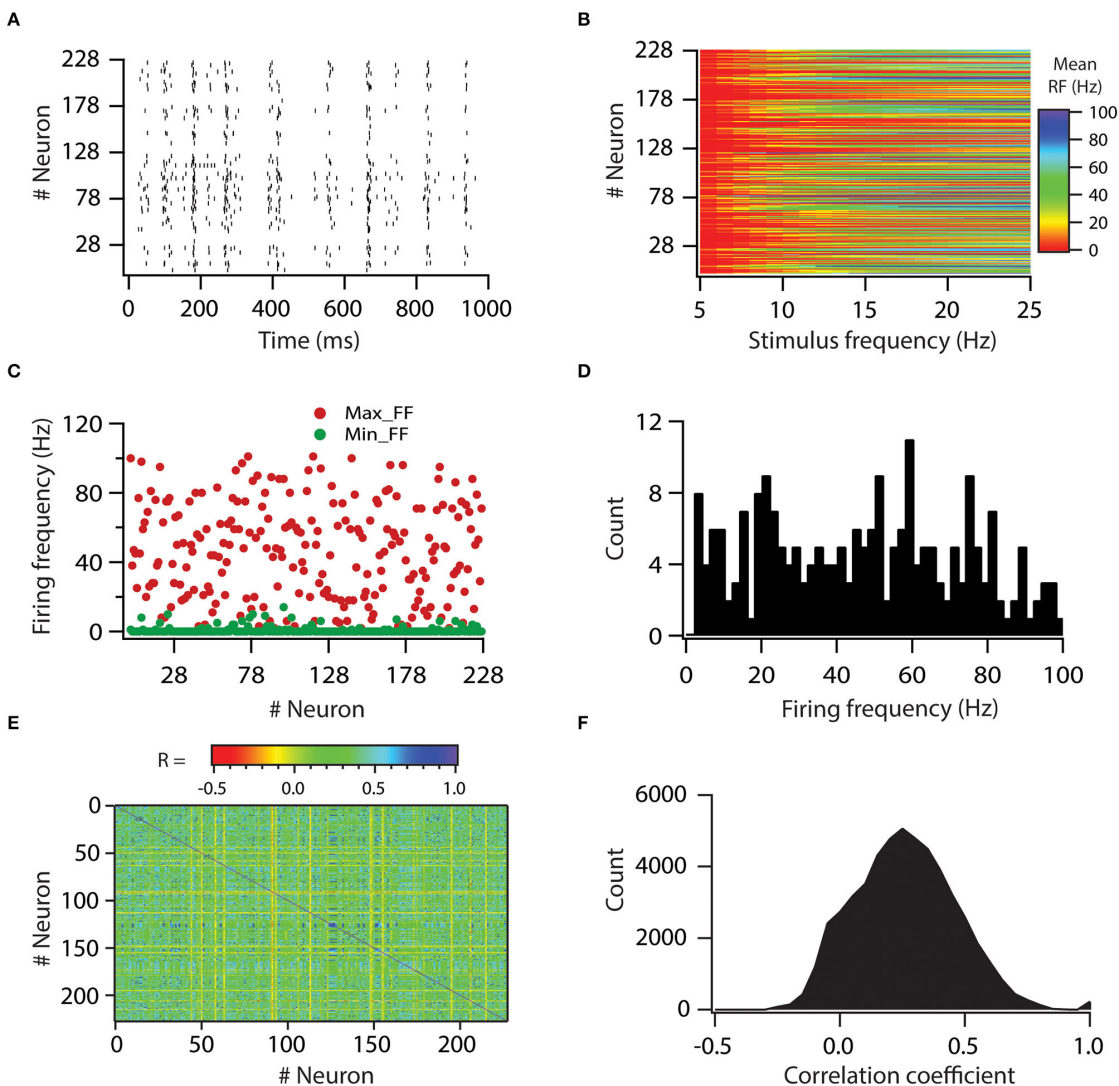
Variability in expression of functional maps leads to decorrelation in firing rates of the neuronal population. **(A)** Spike raster for all the valid model neurons in response to 8-Hz Poisson-distributed synapse activation. A spatio-temporal activation pattern of synapses was same across the neuronal population. **(B)** Color-coded mean firing frequency, averaged over 50 trials, as a function of stimulus frequency. **(C)** Distribution of minimum and maximum firing frequencies in model neurons. **(D)** Histogram of maximum firing frequencies in model neuron population. **(E)** Color-coded matrix of correlation coefficient values for firing frequencies. **(F)** Histogram of correlation coefficient values for firing frequencies in model neuron population. Note that for a given stimulus frequency, a spatio-temporal activation pattern of synapses was trial-matched across the neuronal population.

Afterward, we assessed the role of variability in VGICs expression and passive membrane properties in modulating the variability of neuronal output. Specifically, we aimed to investigate whether variability in neuronal input processing is translated to variability in neuronal output. For this aim, we first generated a raster plot of spike times for all neurons for a given stimulus frequency of 8 Hz (Figure 3A). For this, all synapses were activated in a Poisson manner at once. The controlled input of a spatio-temporal activation pattern of synaptic inputs was kept constant for all neurons. As visible from the graph, the simulation shows that different neurons emit different numbers of spikes for this given stimulus frequency, which suggests that the firing frequency of neurons is variable. Similarly, it is demonstrated that apart from firing frequency, spike timings also show variability (Figure 3A).

Thereafter, we examined the variability in response to the entire physiological range of stimulus frequencies. For that, we computed firing frequencies for all neurons for all stimulus frequencies. Again, a spatio-temporal activation pattern of synaptic inputs was kept constant for all neurons and was trail matched. Thus, any variability incurred in firing frequencies across neurons would be due to variability in VGICs expression and passive membrane properties. Plotting the firing frequencies for all neurons against stimulus frequencies showed that firing frequencies exhibit huge variability across all neurons (Figure 3B). To get a deeper understanding of this variability, we plotted minimum and maximum firing frequencies for all neurons (Figure 3C). From this, we noted that minimum firing frequencies did not show huge variability as most of the neurons did not fire action potentials at minimum stimulus frequency (5 Hz). However, in contrast to minimum firing frequencies, maximum firing frequencies displayed huge variability across neurons, from 2 to 100 Hz (Figures 3C,D). These analyses showed that variability in VGICs expression and passive membrane properties cause large variability in neuronal firing frequencies.

Next, given that correlation among firing frequencies between neuronal pairs could have a profound effect on information encoding (Panzeri et al., 1999), we investigated how variability in VGICs expression and passive membrane properties would affect this correlation of firing frequencies across the neuronal pairs. When we generated average correlation coefficient values among all possible pairs of neurons across the range of stimulus frequencies, we found that the average correlation coefficient values were generally small (Figures 3E,F) in the range of −0.2 to 0.5. Only a subset of neuronal pairs had average correlation coefficient values >0.5. This analysis demonstrates that variability in VGICs expression and passive membrane properties enables the decorrelation in firing frequencies among the neuronal pairs.

### Variability in Voltage-Gated ion Channel Expression Causes Variable Information Encoding Capabilities

Next, we determined whether the variability in input and output processing impacts the information encoding capabilities of neurons. For that, we employed a rate coding schema, and by using a well-established Shannon entropy principle, we computed mutual information of individual neurons. In this scenario, a synaptic activation at different stimulus frequencies forms the incoming information which is subsequently encoded by neurons' output firing frequencies. In this system, mutual information is defined as the capability of the neuron to successfully encode or represent different stimulus frequencies by the variability of the neuron's output firing frequencies. In such a system, a synaptic activation pattern was Poisson-distributed spike trains at different stimulus frequencies.

**Figure 4:**
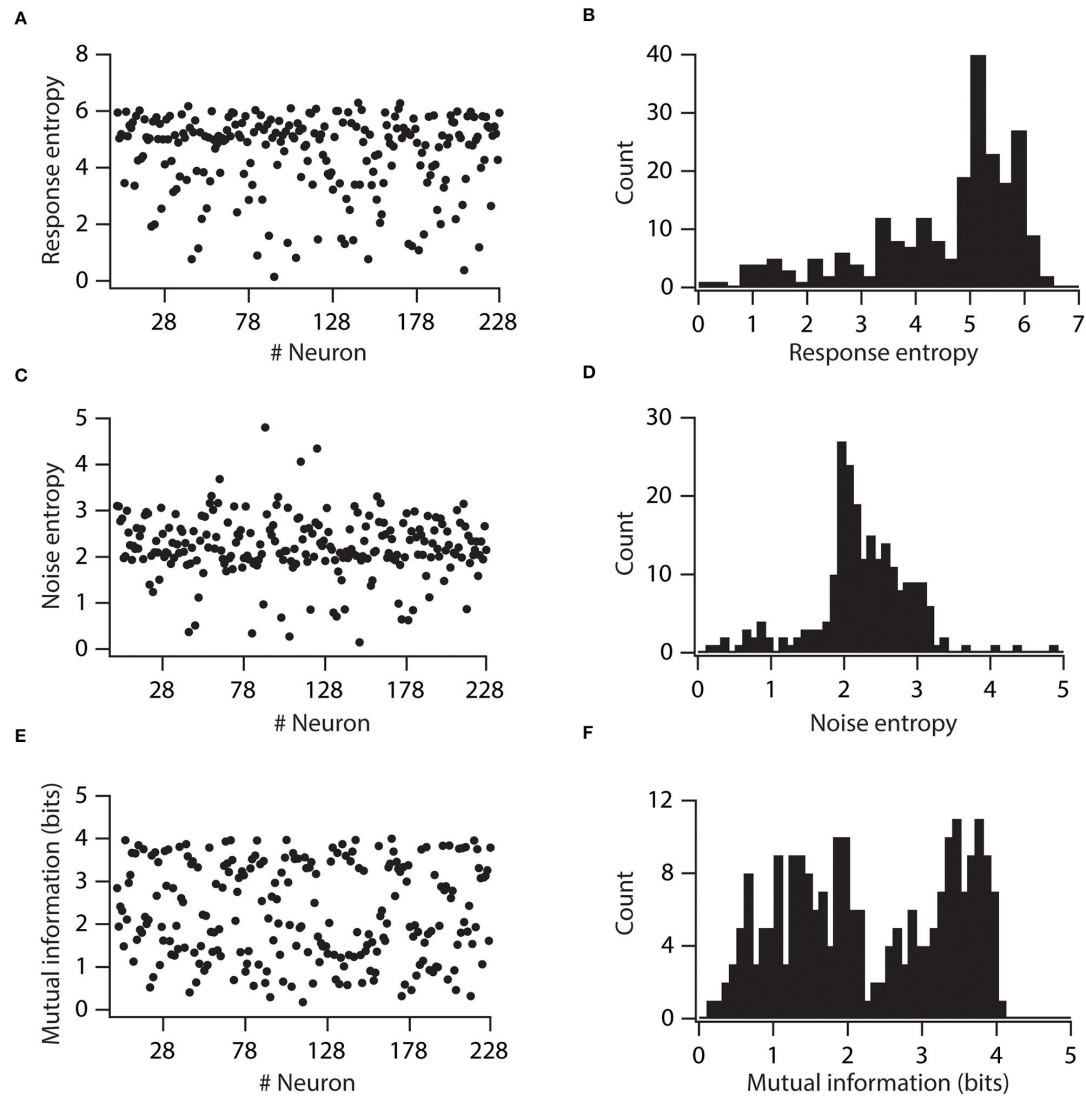
Variability in expression of functional maps leads to distributed mutual information in the neuronal population. **(A)** Total response entropy for all the valid neurons. **(B)** Distribution of total response entropy in the population of valid neurons. **(C)** Noise entropy for all the valid neurons. **(D)** Distribution of noise entropy in the population of valid neurons. **(E)** Mutual information for all the valid neurons. **(F)** Distribution of mutual information in the population of valid neurons.

First, we computed response entropy for the given range of stimulus frequencies (5 to 25 Hz) for individual neurons. With this, we found that response entropies of individual neurons displayed large variability (Figures 4A,B). Similarly, the noise entropy of individual neurons also showed variability (Figures 4C,D). Mutual information was then calculated by subtracting noise entropy from response entropy. We found that the mutual information of individual neurons showed huge variability across the neuronal population (Figures 4E,F). This neuronal population spanned the spectrum of mutual information from as low as 0.1761 bits to as high as 4.02 bits. These analyses demonstrated that variability in VGICs expression and passive membrane properties causes the variability in information encoding capabilities of neurons.

**Figure 5:**
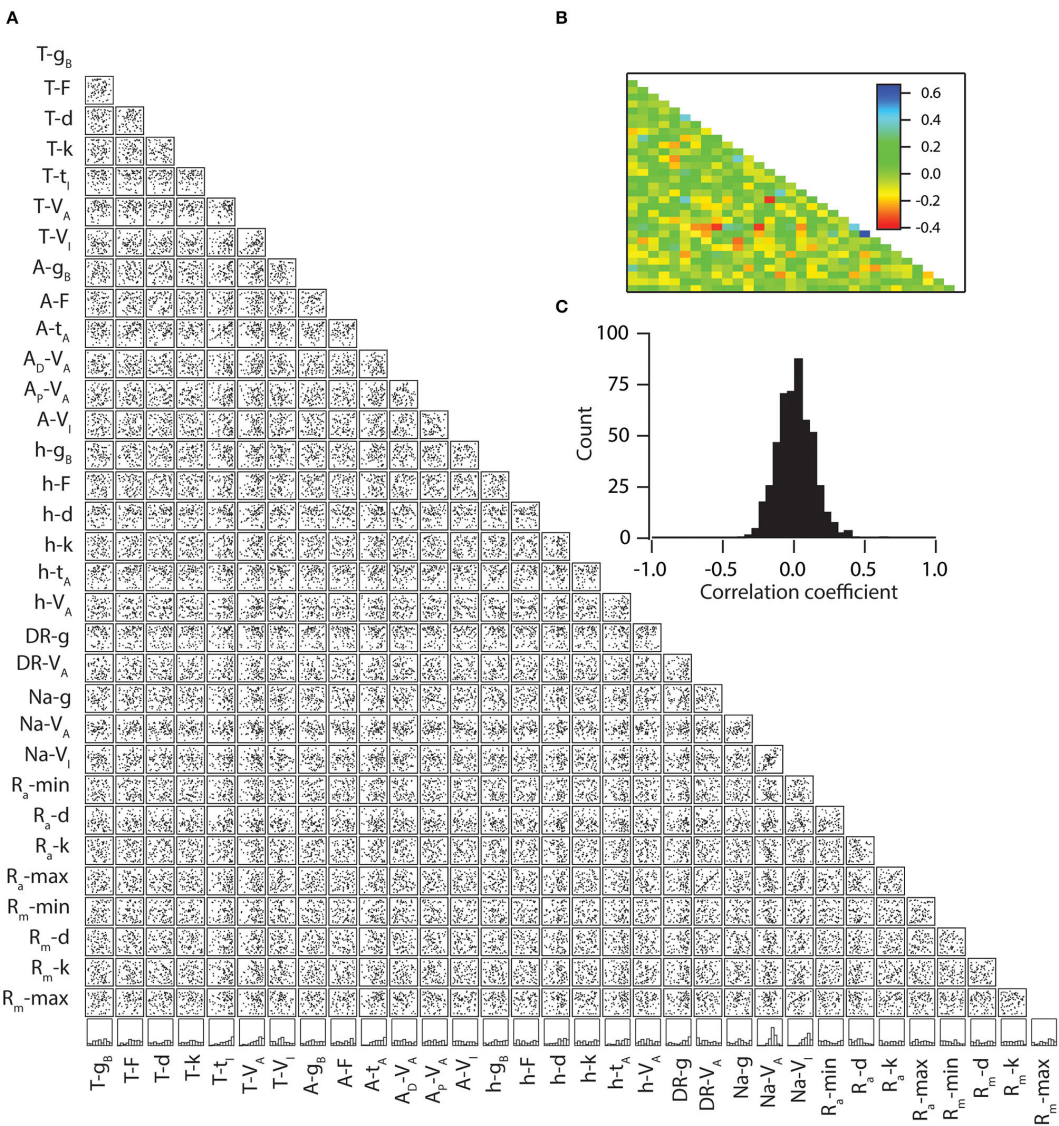
Weak pair-wise correlations between parameters for the models with similar mutual information. **(A)** Lower diagonal of a matrix depicting interactions among the 32 parameters derived from all valid models (*n* = 80). Each subpanel depicts a scatter plot of the values of two parameters (labeled below and left) derived from all valid models. Correlation coefficients were computed for each of the scatter plots. The bottom-most row denotes the normalized histograms of individual parameters in the models with similar mutual information. **(B)** Lower diagonal of a color-coded matrix of correlation coefficients corresponding to the scatter plots in **(A)**. **(C)** Distribution of correlation coefficients for the 496 pairs corresponding to the scatter plots in **(A)**.

Following, we explore the underlying principle that determines the parameters that enable the high and low information encoding capabilities of neurons. Specifically, we asked whether neurons with high mutual information transfer capabilities have any kind of preference toward any distinct parametric space for each parameter. To do this, we selected neurons whose mutual information was in the range of >3 bits, which yielded 80 neuronal models. Next, we generated histograms of all 32 parameters of the 80 neuronal models (Figure 5A, bottom-most row). This analysis showed that all the parameters were spanning through their entire range. To conclude, we observed no restriction on the parametric space of the 32 tested parameters that cause high mutual information, at least for the tested range.

Because a previous study showed that morphology and connected nature of compartments were insufficient for inducing a high correlation among the parameters when the population was sampled based upon intrinsic properties, we tested whether high mutual information capabilities enforce a significant correlation between parameters. For that, we took the aforementioned 80 neuronal models and performed linear correlations among their parameters (Figure 5A). We found that the correlation coefficient values were relatively low (>-0.3 and <0.4) among all of the possible pairs of parameters (Figures 5B,C), suggesting that collective channelostasis is the mechanism underlying robust information transfer.

### Virtual Knockout Models Suggest KA Channels Are Major Regulators

**Figure 6:**
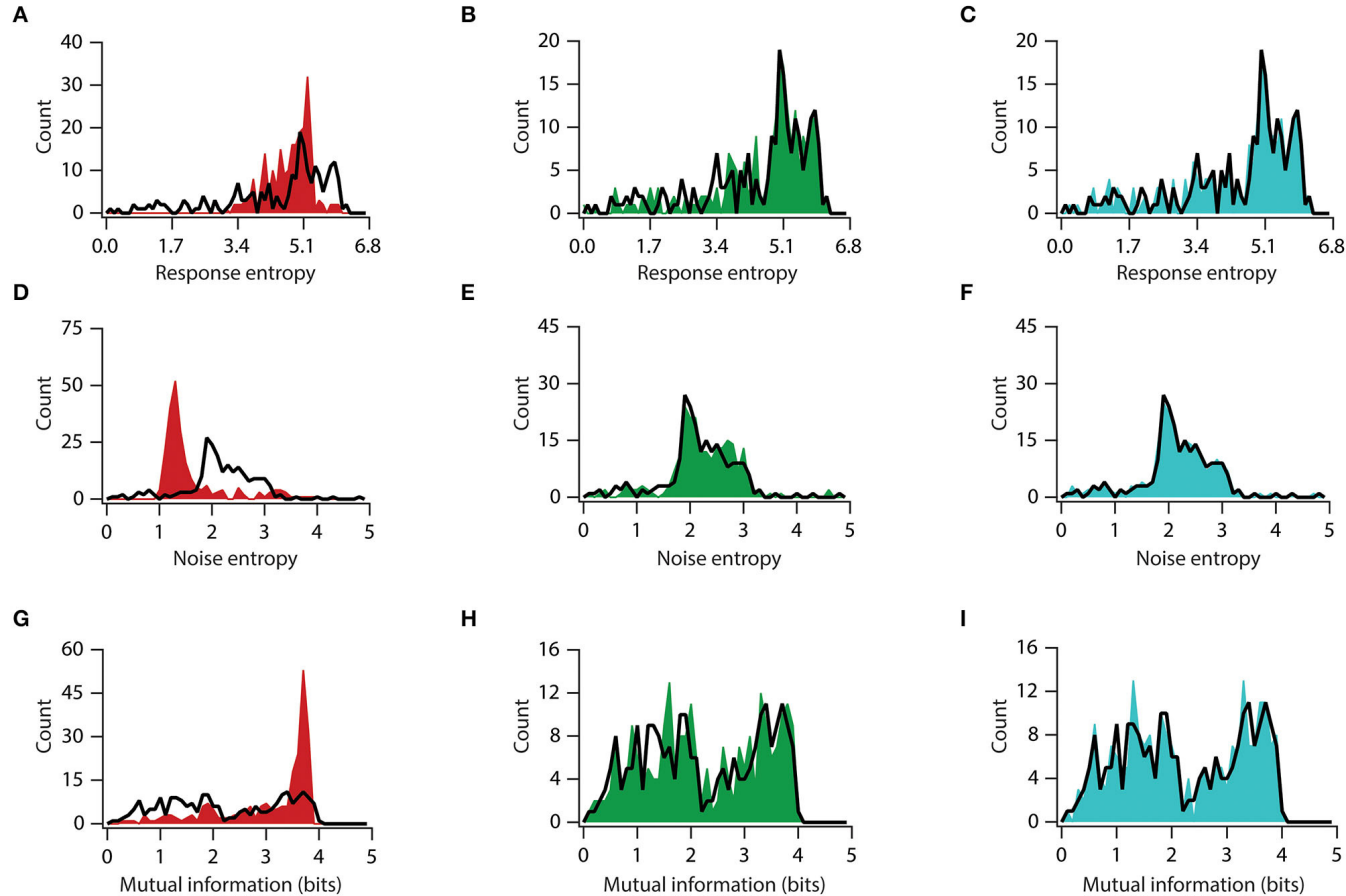
Knocking out conductance has variable impact on mutual information. **(A–C)** Distribution of total response entropy in valid neuronal population (black trace) and virtual knockout model population of KA **(A)**, HCN **(B)**, and CaT **(C)**. **(D–F)** Distribution of noise entropy in valid neuronal population (black trace) and virtual knockout model population of KA **(D)**, HCN **(E)**, and CaT **(F)**. **(G–I)** Distribution of mutual information in valid neuronal population (black trace) and virtual knockout model population of KA **(G)**, HCN **(H)**, and CaT **(I)**.

Within the framework of the herein modeling, we examined whether one of the channels is a major contributor in determining the effects on mutual information. To answer this, we utilized a previously derived virtual knockout methodology (VKM) technique on our model neurons. Specifically, we removed a specific conductance from each of the 228 valid models and repeated our simulations on uEPSP, uIPSP, firing frequency, and mutual information. Given that fast Na^+^ channels and delayed rectifier K+ channels are basic requirements for action potential generation, we used virtual knockout models (VKMs) of only three types of channels: *A*-type K^+^, *T*-type Ca^++^, and HCN channels. These analyses demonstrated the impact of knocking out the specific conductance of either *A*-type K^+^, *T*-type Ca^++^, or HCN channels on information processing and transfer (Figure 6, Supplementary Figures S3, S4).

At first, we looked at the role of *A*-type K^+^, *T*-type Ca^++^, and HCN channels in affecting input processing. In accordance with the VKM, we removed each time the specific conductance related to either *A*-type K^+^ or *T*-type Ca^++^ or HCN channels and simulated the uEPSP and uIPSP responses from each of the three VKMs. Next, we generated histograms of uEPSPs and uIPSPs and compared the histograms of the VKMs responses to the responses histograms of the baseline valid (BLV) neurons (Supplementary Figure S2). These comparisons show that the removal of *A*-type K^+^ channels led to an increase in uEPSP and uIPSP amplitude (Supplementary Figure S3). Specifically, removing *A*-type K^+^ channels increased the mean uEPSP response from 0.192 to 0.357 mV (D = 0.55, *p* < 0.0001, in Kolmogorov–Smirnov [K–S] between *A*-type K^+^-KO and BLV neuron) and the response of the uIPSP increased from a mean value of −1.01–−1.19 mV (D = 0.46, *p* < 0.0001, in K–S between *A*-type K^+^ KO and BLV neuron). Removing HCN channels did not alter the uEPSP response (0.192–0.201 mV) (D = 0.20, *p* = 0.34, in K–S between HCN-KO and BLV neuron) and the uIPSP response (−1.01–−1.05 mV) (D = 0.09, *p* > 0.99, in K–S between HCN-KO and BLV neuron). Similarly, removing *T*-type Ca^++^ channels did not change the uEPSP (0.192 mV to 0.1.85 mV) (D = 0.10, *p* > 0.99, in K–S between *T-type* Ca^2+^-KO and BLV neuron) and the uIPSP amplitude (−1.01–−1.00 mV) (D = 0.09, *p* > 0.99, in K–S between *T-type* Ca^2+^-KO and BLV neuron). These analyses showed that only the removal of *A*-type K^+^ channel-specific conductance had an impact on input processing in the neuronal population.

Next, we analyzed the role of *A*-type K^+^, *T*-type Ca^++^, and HCN channels in affecting output processing. To this end, we generated the input/output relationship of all valid models after removing the specific conductance of either *A*-type K^+^, *T*-type Ca^++^, or HCN channels and compared the resultant firing frequency to that of the baseline valid model population. These analyses showed that the removal of *A*-type K^+^ channels results in an increased firing frequency (Figure 4SA), from a mean firing frequency of neurons of 26.78 Hz in the baseline model to 49.57 Hz after removing *A*-type K^+^ conductance (D = 0.31, *p* = 0.0001, in K–S between *A*-type K^+^ KO and BLV neuron). In contrast, the removal of either HCN or *T*-type Ca^2+^ channels did not change the firing frequency outputs distribution and mean frequencies (28.59 and 26.76 Hz for HCN-KO and *T*-type Ca^2+^-KO, respectively; D = 0.12, *p* = 0.47, and D = 0.04, *p* > 0.99, in K–S between HCN-KO and BLV neurons and between *T*-type Ca^2+^-KO and BLV neurons, respectively) (Figures 4SB,C). In conclusion, among the channels we examined, only *A*-type K^+^ conductance is a significant contributor toward constraining neuronal firing frequency.

Next, we examined each channel's contribution to information transfer. For this, we used a similar approach of analyzing elements of information transfer after removing each specific conductance. An analysis of response entropy in model neurons showed that the removal of the *A*-type K^+^ channels significantly altered the distribution of response entropy, with a significant increase in mean response entropy from 4.5 to 4.8 bits (t_(454)_ = 2.93, *p* < 0.01, for unpaired *t*-test; D = 0.40, *p* < 0.0001, in K–S between *A*-type K^+^ KO and BLV neurons) (Figure 6A), while the removal of the HCN or *T*-type Ca^2+^ conductance did not affect the response entropy distribution or their means (t_(454)_ = 0.87, *p* = 0.38, for unpaired *t*-test and D = 0.05, *p* > 0.99; t_(454)_ = 0.15, *p* = 0.89, for unpaired *t*-test and D = 0.08, *p* = 0.99, in K–S between HCN*-*KO and BLV neurons and between *T*-type Ca^2+^-KO and BLV neurons, respectively) (Figures 6B,C). On the other hand, when we looked into noise entropy, we found that removing *A*-type K^+^ channels decreased the noise entropy in model neurons, with a mean noise entropy reduction from 2.20 to 1.68 (t_(454)_ = 7.97, *p* < 0.0**00**1, for unpaired *t*-test), although the distribution was not significantly altered (D = 0.31, *p* = 0.17, in K–S) (Figure 6D). However, the removal of HCN and *T*-type Ca^2+^ channels did neither alter the means nor the distributions of noise entropy (t_(454)_ = 1.13, *p* = 0.26, and t_(454)_ = 0.22, *p* = 0.83, in unpaired *t*-tests; D = 0.11, *p* > 0.99, and D = 0.04, *p* > 0.99, in K–S, between HCN*-*KO and BLV neurons and between *T*-type Ca^2+^-KO and BLV neurons, respectively, for each test) (Figures 6E,F). Next, we analyzed mutual information and found that removing *A*-type K^+^ channels improved the mutual information, with mean mutual information increasing from 2.28 bits to 3.10 bits (t_(454)_ = 8.63, *p* < 0.0001, for unpaired *t*-test; D = 0.35, *p* < 0.05, in K–S between *A*-type K^+^-KO and BLV neurons) (Figure 6G). In contrast, removing *T*-type Ca^++^ and HCN channels did not affect mutual information (t_(454)_ = 0.37, *p* = 0.71, for unpaired *t*-test and D = 0.08, *p* > 0.99; t_(454)_ = 0.05, *p* = 0.96, for unpaired *t*-test and D = 0.05, *p* > 0.99, in K–S between HCN*-*KO and BLV neurons and between *T*-type Ca^2+^-KO and BLV neurons, respectively) (Figures 6H,I). Taken together, these analyses showed that the *A*-type K^+^ channel is the major regulator of information transfer at least within the framework of our analyses.

## Discussion

Intricate regulation of information encoding/transfer capabilities is extremely important for the individual neurons, their circuits, and the brain as a whole. Hence, it is essential to elucidate how neurons achieve this regulation, given the fact that they express a rich and differential repertoire of voltage-gated ion channels (VGICs) across the dendrite–soma–axon continuum. The presence of these VGICs has a tremendous influence on the input, integration, and output module of the neuron.

Furthermore, neurons are affected by additional multiple factors, which are given as follows:

1. Intracellular biochemical milieu, upon which cellular processes are heavily dependent and receive continuous perturbations (Marder and Thirumalai, 2002; Desai, 2003; Frick et al., 2004; Turrigiano and Nelson, 2004; Fan et al., 2005).
2. Various ion channels, which define basic neuronal properties undergo continuous trafficking at the plasma membrane (Lai and Jan, 2006; Shepherd and Huganir, 2007; Vacher et al., 2008; Shah et al., 2010; Nusser, 2012).
3. Properties of ion channels are susceptible to change by various factors including phosphorylation/dephosphorylation, interaction with intracellular messengers, and lipid composition of the plasma membrane (Levitan, 1994; Ismailov and Benos, 1995).
4. Continuous rewiring of synaptic connectivity (Chen et al., 2014; Attardo et al., 2015).
5. Changes in dendritic arborization at microscopic (spines) and macroscopic (dendritic branches) levels (Ikegaya et al., 2001; Yuste and Bonhoeffer, 2001; Attardo et al., 2015).
6. Dynamics related to various functions brought about by astrocytes, oligodendrocytes, and microglial cells (Baumann and Pham-Dinh, 2001; Haydon and Carmignoto, 2006; Sierra et al., 2014). Yet, despite all of these ongoing dynamics of perturbations, neurons maintain their stability and functionality and perform robust functions.

In this study, by performing global sensitivity analyses on neuronal information encoding/transfer capabilities, we show that neurons can achieve similar information encoding/transfer capabilities in several non-unique ways (Figure 5). This implies that neural mechanisms, involved in information encoding, e.g., VGICs, need not maintain the density and properties of individual channels at particular specific values (Rathour and Narayanan, 2014). This brings a tremendous opportunity for neurons to encode novel information through several non-unique combinations of ion channels. Therefore, collective channelostasis presents an important answer to the aforementioned question.

In addition, we have demonstrated that the contribution of the various VGICs is highly differential. In our simulations, we showed that between the three VGICs, namely, *A*-type K^+^, HCN, and *T-type* Ca^2+^, the major contribution was of the *A*-type K^+^ channel, and its knockout completely altered the information processing, resulting in a significantly aberrant output (Figure 6, Supplementary Figures 3S, 4S). Investigating the differential contribution of each channel to information encoding has the potential to contribute to a more comprehensive understanding of the effects of various genetic mutations on brain functioning, leading to cognitive and behavioral deficits. Our finding suggests that genetic variations in some VGICs are less lenient, and will cause a much more severe impact on brain functioning than others.

Variability in VGICs and passive membrane properties could be crucial where inputs are highly correlated in a homogeneous neuronal population, which eventually leads to the correlated output spiking (Panzeri et al., 1999). Output correlation could have a severe effect on the information. There are a number of factors that could contribute toward a decrease in correlation. One such factor is variability in VGICs and passive membrane properties. Our analysis with firing rate correlation showed that correlation values among their firing frequencies were generally low (Figure 4), while inputs that were introduced by us were perfectly correlated. Therefore, variability in VGICs and passive membrane properties helps in inducing decorrelation of the output activity. Although we did not explicitly analyze spike times correlation, analyses of the raster plots for all neurons for a given input frequency and trial showed that the neurons firing patterns were phase shifted to each other (Figure 3A). This gave us a glimpse that variability in VGICs and passive membrane properties could help in causing decorrelation in spike times.

Although our model neurons were homeostatically stable with a reference to six coexistent functional maps, the model neurons did not show high mutual information (Figure 4). How do we reconcile this? A possible explanation can be that each neuron is tuned to different optimal variabilities to encode incoming stimuli (Tripathy et al., 2013). Therefore, it stands to reason that during learning these neurons could adjust their level of variability such that they could encode incoming stimuli more efficiently. Further studies should focus on how a different degree of variability affects mutual information and homeostasis.

## Methods

### Model and Valid Neuronal Population

A 3D reconstructed CA1 neuronal morphology was used as a substrate for all the simulations. The base model and valid neuronal population were taken from the previous study (Rathour and Narayanan, 2014). In brief, our model neurons expressed five voltage-gated ion channels (VGICs): fast Na^+^, delayed rectifier K^+^, A-type K^+^, T-type Ca^++^, and HCN channels. A valid neuronal population was generated by performing a global sensitivity analysis on a hand-tuned base model. The base model was hand-tuned in such a way that six coexistent functional maps matched their experimental counterparts. After having the experimentally constrained base model, we randomized 32 parameters, associated with five voltage-gated ion channels and passive membrane properties, within a large neighborhood of its default values, and followed an independent uniform distribution within that range. We generated a population of 20,420 models, with each model built by assigning independently random values for each of the 32 parameters in the base model. After having the model population, we tested the model properties corresponding to an experimental counterpart. Specifically, from the experimental data, we assigned a range for six measurements at three different locations along the somato-apical trunk. Each model neuron's properties were tested against these experimental ranges, and if a model neuron satisfies all the 18 constrains (six measurements at three different locations), it is called a valid neuron. Performing this validation procedure on each model resulted in 228 valid neurons. Owing to the validation procedure, this valid neuronal population of 228 neurons was heavily constrained by the experimental data and expressed variability in well-defined six coexistent functional maps. This valid neuronal population of 228 neurons was used throughout the study.

### Synaptic Inputs

Excitatory synapses were modeled as only AMPA receptor-type conductance as modeled previously (Narayanan and Johnston, 2010). Inhibitory synapses were modeled as only GABA-_A_ receptor-type conductance as modeled previously (Sinha and Narayanan, 2015). Each model neuron was endowed with 327 excitatory and 50 inhibitory synapses. Excitatory synapses were distributed uniformly only on an apical dendrite and in the distance from 12 to 294 μm from the soma. Inhibitory synapses were distributed uniformly only in a perisomatic region. As the model population was generated by randomly assigning the values of 32 parameters, related to five VGICs and passive membrane properties, the number of compartments for neurons in the valid neuronal population was highly variable. Thus, in order to match the synaptic locations across the neurons, all neurons were recompartmentalized such that for a given section, a number of compartments were counted across all the neurons of the population, and then the section was recompartmentalized with the higher most count value, thus insuring the uniformity of numbers of compartments for a given section across all the neurons of valid population. Doing this recompartmentalization on each section yielded a total of 909 compartments for a given neuron of the valid population. A synaptic activation pattern was Poisson-distributed. Excitatory synapses were activated at different frequencies ranging from 5 to 25 Hz in steps of 1 Hz, and each stimulus frequency had 50 trials. Inhibitory synapses were activated only at 5 Hz irrespective of the excitatory synaptic stimulus frequency and were used throughout the study. A spatio-temporal activation pattern of excitatory and inhibitory synapses was trial-matched across the neurons of the valid population.

### Mutual Information

Mutual information between response frequency and stimulus frequency was computed as described previously (Honnuraiah and Narayanan, 2013). Specifically, mutual information was taken to be the difference between total response entropy and noise entropy:


$$\begin{array}{l} I_{m}=H-H_{noise} \end{array}$$

where *I*_m_ is the mutual information, *H* is the total response entropy, and *H*_noise_ is the noise entropy. The total response entropy, *H*, was computed as follows:


$$\begin{array}{l} H=-\underset{r}{\sum }p\left[r\right]\;\text{lo}{\text{g}}_{2}\text{(p}\left[\text{r}\right]\text{)} \end{array}$$

where p[r] denotes the response probability distribution of response frequency, r, over the entire range of stimulus frequencies (see synaptic inputs section). The response probability distribution of response frequency was computed as follows:


$$\begin{array}{l} p\left[r\right]=\underset{s}{\sum }p\left[s\right]\;\text{p}\left[\text{r}\;|\text{s}\right]\; \end{array}$$

where p[r|s] denotes the response probability distribution of response frequency, r, for a given stimulus frequency, s. p[s] was assumed to be uniformly distributed as the presentation of any stimulus frequency was equally probable. p[r|s] was computed from the array containing response frequency values for 50 trials for a given stimulus frequency. The first- and second-order statistics of an array containing response frequency values for 50 trials for a given stimulus frequency were used to generate p[r|s] with an implicit assumption of a normal distribution for p[r|s].

In order to compute noise entropy, the entropy of the responses for a given stimulus, s, was computed:


$$\begin{array}{l} H_{s}=-\underset{r}{\sum }p\left[r|s\right]\;\text{lo}{\text{g}}_{2}\left(p\left[r|s\right]\right) \end{array}$$

and then, noise entropy was computed as follows:


$$\begin{array}{l} H_{noise}=\underset{s}{\sum }p\left[s\right]H_{s} \end{array}$$

## Computational Details

All simulations were performed using the NEURON simulation environment (Carnevale and Hines, 2006) at −65 mV, and a temperature was set at 34° C, which accounted for ion channel kinetics relative to their q10 values. For solving various differential equations, an integration time step was set at 25 μs. All analyses were performed using custom-built software written with IGOR Pro (WaveMetrics Inc., USA). A correlation analysis was performed using the statistical computing package *R* (http://www.R-project.org).

## Data Availability Statement

Publicly available datasets were analyzed in this study. This data can be found here: http://neuromorpho.org/.

## Author Contributions

RKR and HK designed the research, analyzed the data, and wrote the paper. RKR performed the research. HK supervision and funding acquisition. Both authors contributed to the article and approved the submitted version.

## Funding

This work was supported by the Israel Science Foundation, Grant Number 248/20 (HK).

## Conflict of Interest

The authors declare that the research was conducted in the absence of any commercial or financial relationships that could be construed as a potential conflict of interest.

## Publisher's Note

All claims expressed in this article are solely those of the authors and do not necessarily represent those of their affiliated organizations, or those of the publisher, the editors and the reviewers. Any product that may be evaluated in this article, or claim that may be made by its manufacturer, is not guaranteed or endorsed by the publisher.

## Supplementary Material

Figure S1 Validation of model population using firing rate profiles. (A) Firing rate profiles of model neurons (red dots) and neurons from experiments (black dots) for different current injections. (B) Same as A. But data is presented as mean ± SEM.

Figure S2 Synaptic permeability values and corresponding somatic PSP potentials in base models. (A-D) Somatic EPSP (A) and IPSP (C) amplitudes and their corresponding synaptic permeability values for excitatory (B) and inhibitory (D) synapses.

Figure S3 Effects of removing a specific voltage gated ion channels on PSP potentials in a population of valid model neurons. (A & D) Removal of A-type K^+^ channels causes an increase in EPSP (A) and IPSP (D) amplitudes. (B & E) Removal of h channels causes a non-significant trend for an increase increase in EPSP (B) and IPSP (E) amplitude. (C & F) Removal of T-type Ca^+^ channels does not affect EPSP (C) and IPSP (F) amplitude.

Figure S4 Effects of removing a specific voltage gated ion channels on firing frequency in a population of valid model neurons. (A) Removal of A-type K^+^ channels causes an increase in firing frequency. Removal of either h channels (B) or T-type Ca^+^ channels (C) does not affect firing frequency.

Figure S5 Effect of removing a specific voltage gated ion channels on firing frequency correlation in a population of valid model neurons. (A-B) Removal of A-type K^+^ channels causes an increase in firing frequency correlation. Removal of either h channels (C-D) or A-type K^+^ channels (E-F) does not affect firing frequency correlation.
