# Supplementary material for: Voltage-Gated Ion Channels and the Variability in Information Transfer
Source: Front Cell Neurosci. 2022 Jul 22;16:906313. doi: 10.3389/fncel.2022.906313 (PMC9352938; doi:10.3389/fncel.2022.906313)
Supplement: Supplementary file 1 [file Data_Sheet_1.pdf]

Table S1: Parameters of randomized neuron models, default values and testing ranges

Count	Parameter	Symbol	Default value	Testing range
***R_m* distribution**				
1	Max value (kΩ.cm^2)	R_m-*max*	145	125 to 165
2	Min value (kΩ.cm^2)	R_m-*min*	125	105 to 145
3	Half–maximal point of R_m sigmoid (µm)	R_m-*d*	320	290 to 350
4	Slope of R_m sigmoid (µm)	R_m-*k*	40	20 to 60
***R_a* distribution**				
5	Max value (Ω.cm)	R_a-*max*	110	90 to 130
6	Min value (Ω.cm)	R_a-*min*	10	5 to 15
7	Half–maximal point of R_a sigmoid (µm)	R_a-*d*	320	300 to 340
8	Slope of R_a sigmoid (µm)	R_a-*k*	14	10 to 20
***h* channel properties**				
9	Maximal conductance (µS/cm^2)	h-g_B	40	30 to 55
10	Fold increase	*h-F*	20	15 to 25
11	Half–maximal point of g_h sigmoid (µm)	*h-d*	370	330 to 410
12	Slope of g_h sigmoid (µm)	*h-k*	14	10 to 20
13	$V_{1/2}$ activation of I_h (mV)	h-V_A	–82	–75 to –90
14	Activation time constant of I_h (ms)	h-τ_A	33.089	25 to 75
***A*-type K$^+$ channel properties**				
15	Maximal conductance (mS/cm^2)	A-g_B	3.1	2.6 to 3.7
16	Fold increase per 100 µm	*A-F*	5	4 to 6
17	$V_{1/2}$ activation KA_{prox} (mV)	A_P-V_A	11	5 to 15
18	$V_{1/2}$ activation KA_{dist} (mV)	A_D-V_A	–1	–5 to 5
19	$V_{1/2}$ inactivation KA (mV)	A-V_I	–56	–60 to –50
20	Activation time constant KA (ms)	A-τ_A	0.032	0.02 to 0.1
**Fast Na$^+$ channel properties**				
21	Maximal conductance (mS/cm^2)	*Na-g*	12.5	11 to 14
22	$V_{1/2}$ activation (mV)	Na-V_A	–38	–30 to –45
23	$V_{1/2}$ inactivation (mV)	Na-V_I	–50	–40 to –60
**Delayed rectified K$^+$ channel properties**				
24	Maximal conductance (mS/cm^2)	*DR-g*	10	7 to 13
25	$V_{1/2}$ activation (mV)	DR-V_A	13	5 to 20
***T*-type Ca^{++} channel properties**				
26	Maximal conductance (µS/cm^2)	T-g_B	55	40 to 70
27	Fold increase	*T-F*	25	20 to 30
28	Half–maximal point of g_{CaT} sigmoid (µm)	*T-d*	370	330 to 410
29	Slope of g_{CaT} sigmoid (µm)	*T-k*	15	5 to 25
30	$V_{1/2}$ activation (mV)	T-V_A	–60	–50 to –70
31	$V_{1/2}$ inactivation (mV)	T-V_I	–85	–75 to –95
32	Inactivation time constant (ms)	T-τ_I	31.012	10 to 50
