# Supplementary figures and images for: Voltage-Gated Ion Channels and the Variability in Information Transfer
Source: Front Cell Neurosci. 2022 Jul 22;16:906313. doi: 10.3389/fncel.2022.906313 (PMC9352938; doi:10.3389/fncel.2022.906313)

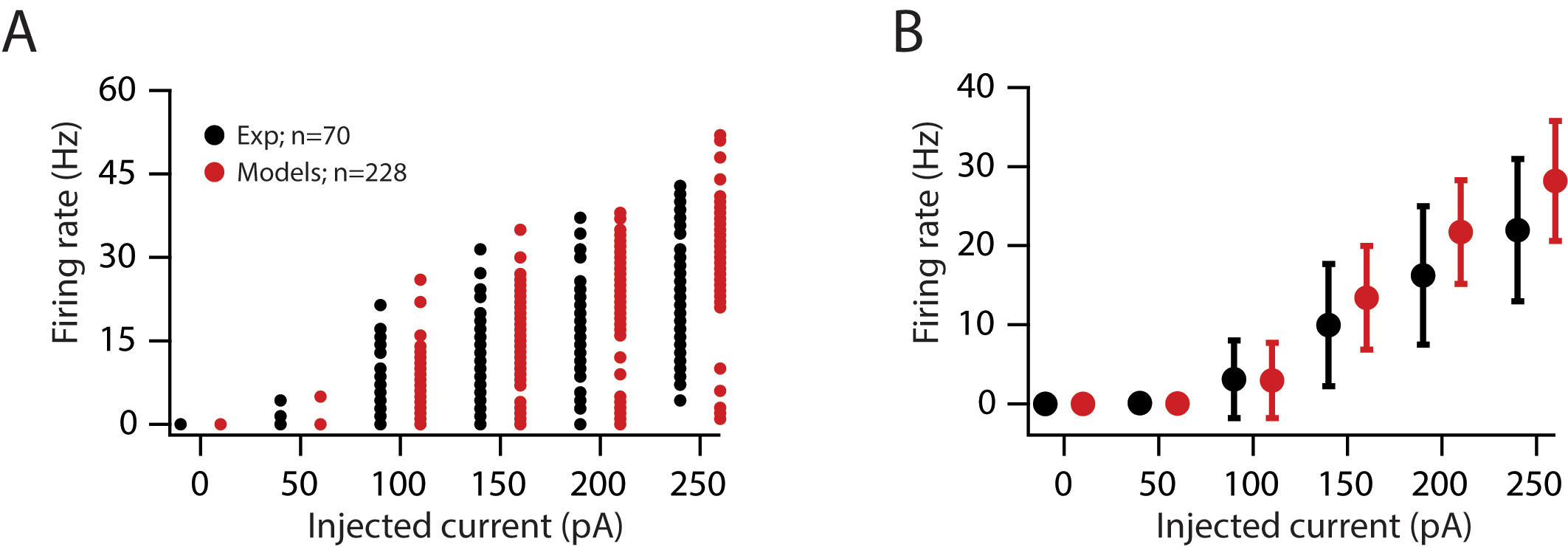

Supplement: Figure S1 — Validation of model population using firing rate profiles. (A) Firing rate profiles of model neurons (red dots) and neurons from experiments (black dots) for different current injections. (B) Same as A. But data is presented as mean ± SEM. [file Image_1.TIF]

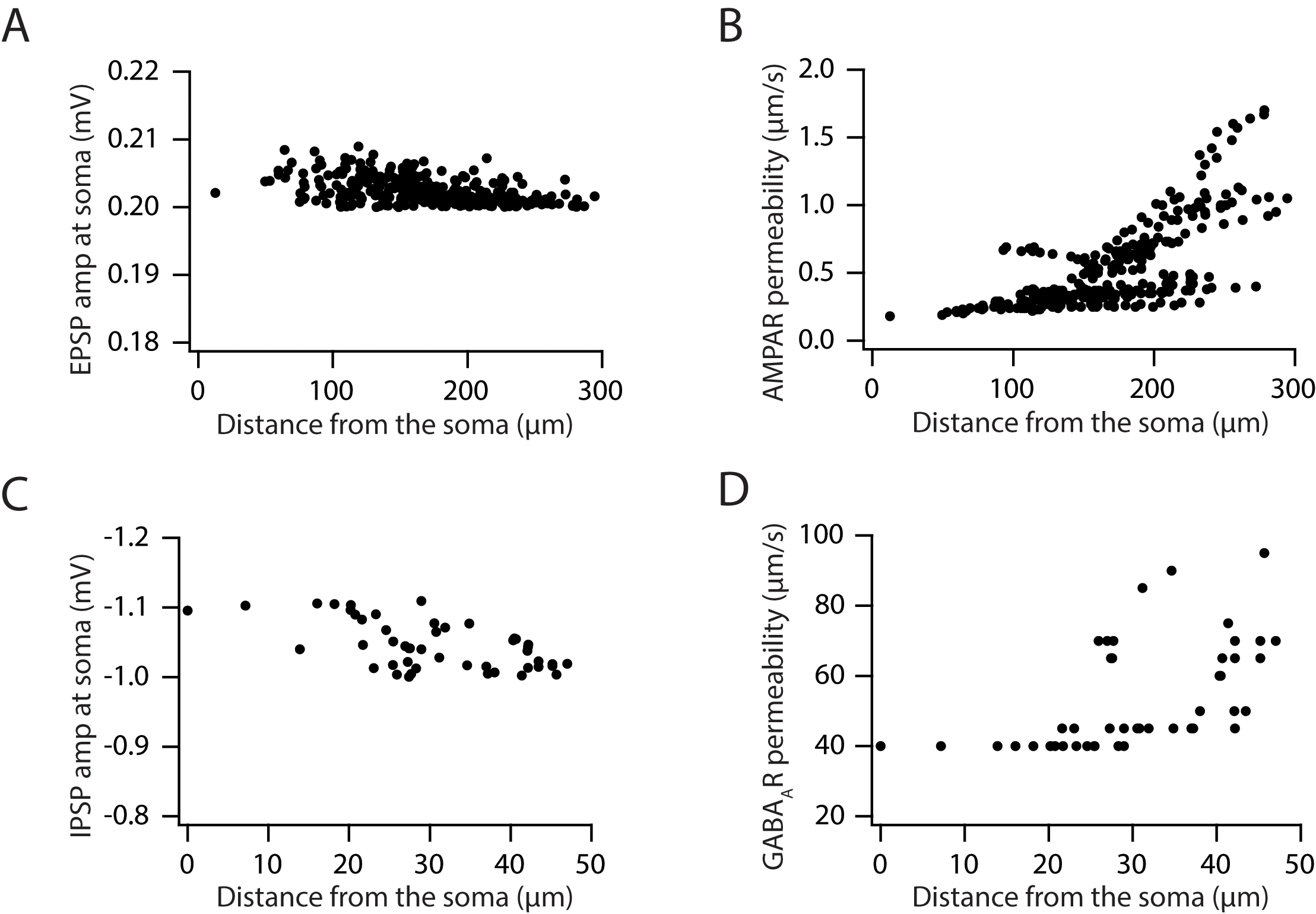

Supplement: Figure S2 — Synaptic permeability values and corresponding somatic PSP potentials in base models. (A-D) Somatic EPSP (A) and IPSP (C) amplitudes and their corresponding synaptic permeability values for excitatory (B) and inhibitory (D) synapses. [file Image_2.TIF]

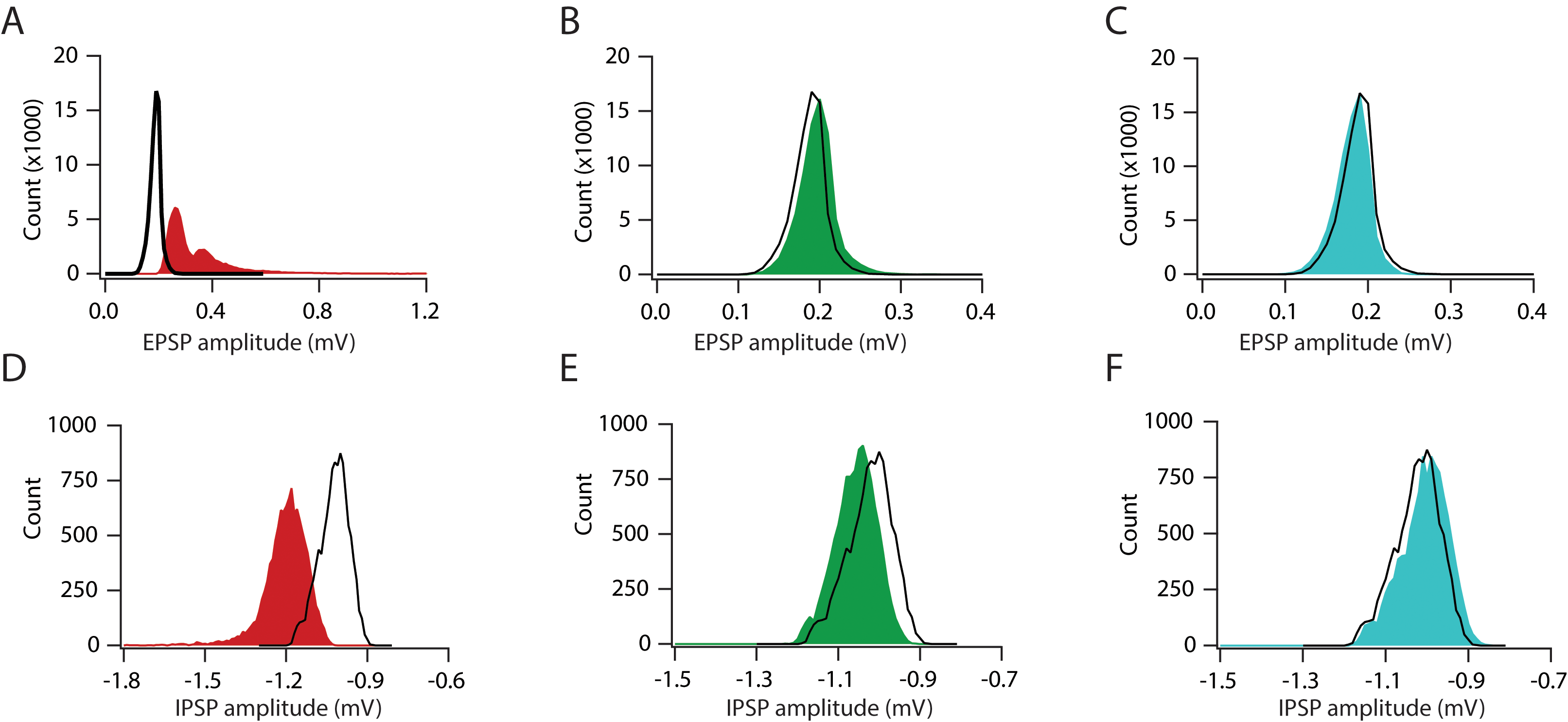

Supplement: Figure S3 — Effects of removing a specific voltage gated ion channels on PSP potentials in a population of valid model neurons. (A & D) Removal of A-type K+ channels causes an increase in EPSP (A) and IPSP (D) amplitudes. (B & E) Removal of h channels causes a non-significant trend for an increase increase in EPSP (B) and IPSP (E) amplitude. (C & F) Removal of T-type Ca+ channels does not affect EPSP (C) and IPSP (F) amplitude. [file Image_3.TIF]

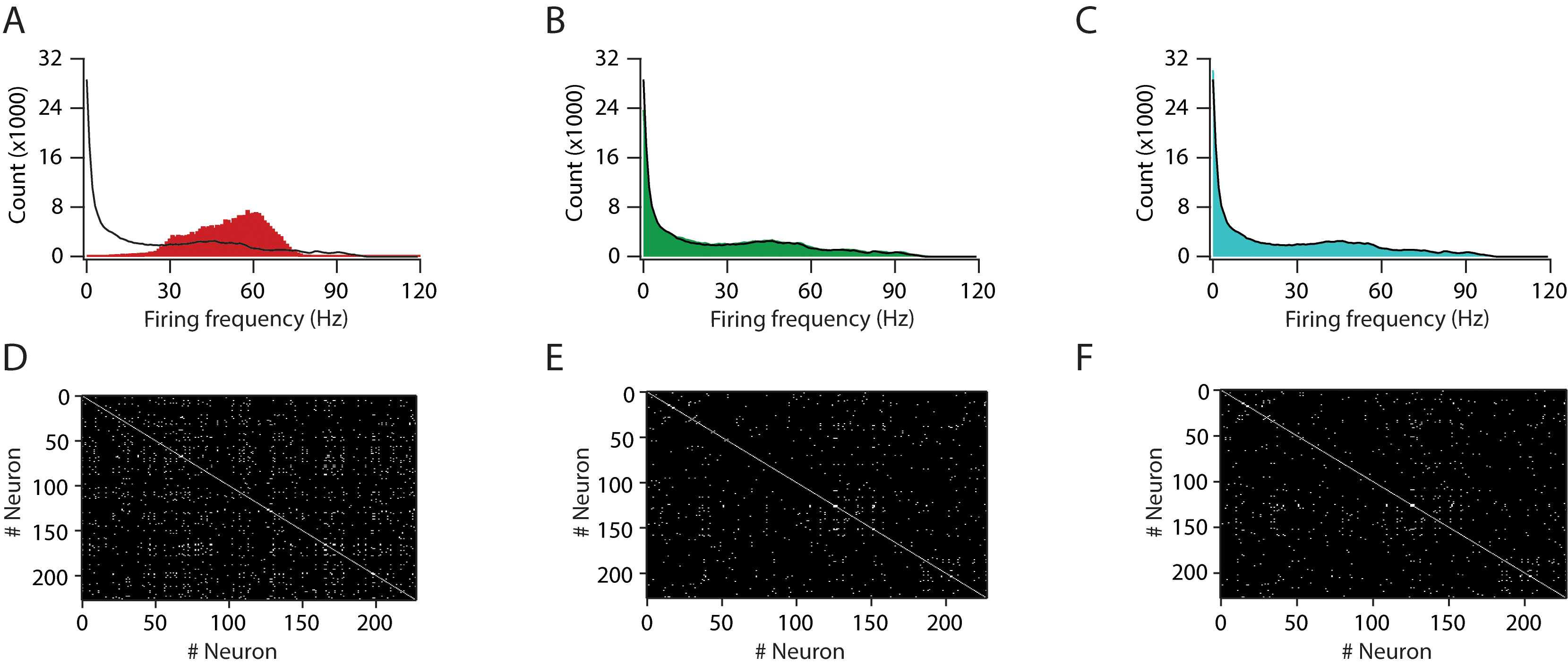

Supplement: Figure S4 — Effects of removing a specific voltage gated ion channels on firing frequency in a population of valid model neurons. (A) Removal of A-type K+ channels causes an increase in firing frequency. Removal of either h channels (B) or T-type Ca+ channels (C) does not affect firing frequency. [file Image_4.TIF]

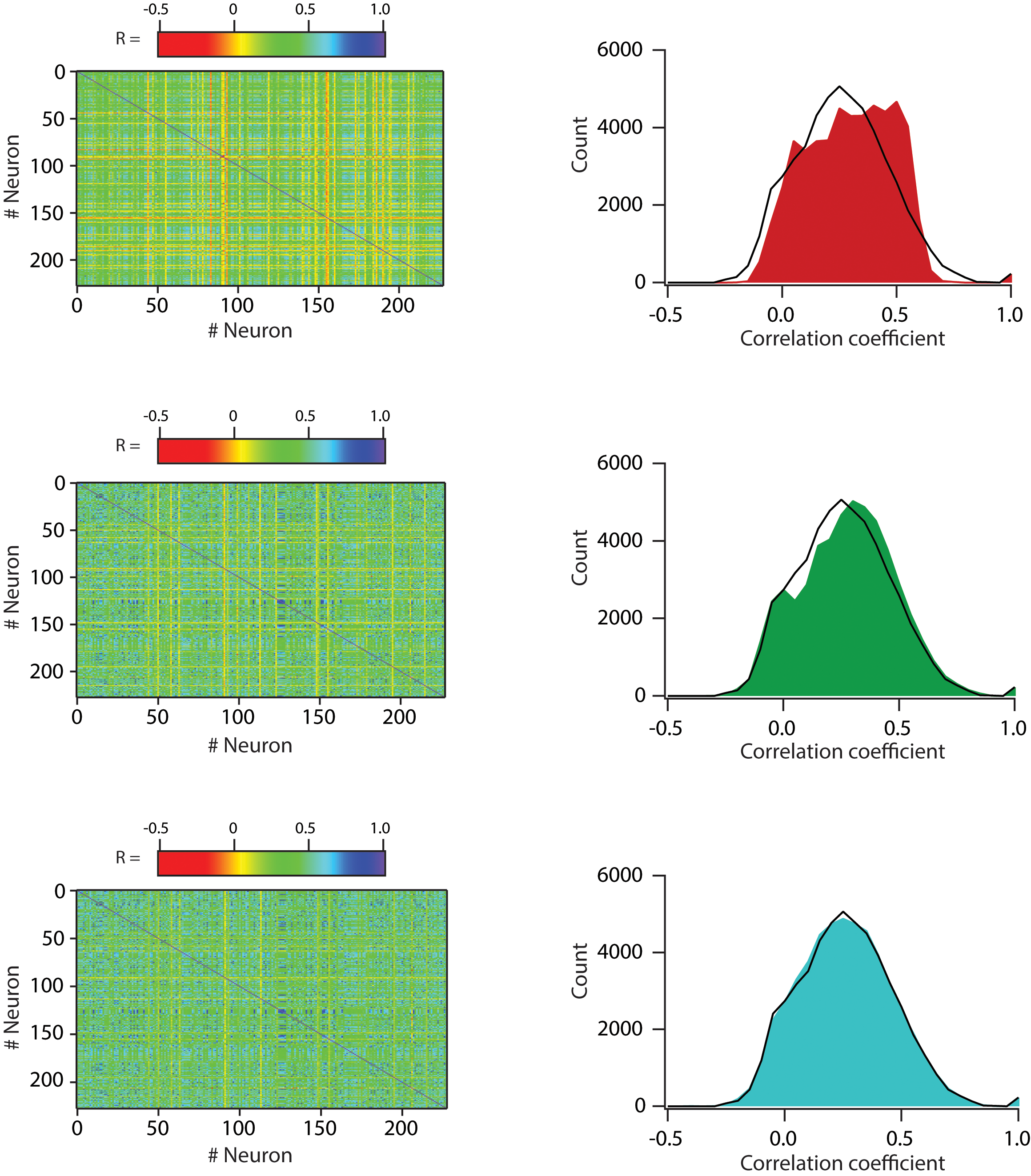

Supplement: Figure S5 — Effect of removing a specific voltage gated ion channels on firing frequency correlation in a population of valid model neurons. (A-B) Removal of A-type K+ channels causes an increase in firing frequency correlation. Removal of either h channels (C-D) or A-type K+ channels (E-F) does not affect firing frequency correlation. [file Image_5.TIF]
